# Standardizing the Production and Processing of Bacterial Nanocellulose to Produce Microparticles via a Spray-Dried Method with the Potential to Be Used in the Food Industry

**DOI:** 10.3390/polym17162193

**Published:** 2025-08-11

**Authors:** Hasbleidy Palacios-Hinestroza, Carlos Molina-Ramírez, María Camila López-Jaramillo, Julián Paul Martínez-Galán, Diego Mauricio Sánchez-Osorno

**Affiliations:** 1 Department of Basic Sciences, Campus Tlajomulco, University of Guadalajara, Tlajomulco de Zúñiga 45641, Mexico; hasbleidy.palacios@academicos.udg.mx; 2 Research Group in Chemistry and Bioprospecting of Natural Products, University of Magdalena, Santa Marta 470003, Colombia; cmolinar@unimagdalena.edu.co; 3 Grupo de Investigación e Innovación Ambiental (GIIAM), Institución Universitaria Pascual Bravo N 73a-226, Medellín 050034, Colombia; m.lopezja@pascualbravo.edu.co; 4 Grupo de Investigación Alimentación y Nutrición Humana-GIANH, Escuela de Nutrición y Dietética, Universidad de Antioquia, Cl. 67, No 53-108, Medellín 050034, Colombia; julian.martinez@udea.edu.co; 5 Grupo de Investigación, QUALIPRO, Institución Universitaria Pascual Bravo, Cl. 73, No 73a-226, Medellín 050034, Colombia

**Keywords:** bacterial nanocellulose, agro-industrial waste, mechanical processing, *Komagataeibacter medellinensis*

## Abstract

This article proposes a standard protocol to produce bacterial nanocellulose (*Komagateibacter medellinensis*). It will briefly review the main raw materials (common agro-industrial waste in tropical countries), process of obtaining bacterial nanocellulose membranes, and the cleaning process for said membranes. The processing of the membranes using a grinder and Ultra-Turrax is then shown, listing the characteristics provided by each of these methods to produce bacterial nanocellulose microparticles by spray drying. The average microparticle size obtained by spray drying was (4.76 ± 1.12 µm), and thermal stability (maximum degradation temperature) was 345 °C. This research clearly states that the grinder was selected as the most efficient mechanical method due to its improved dispersion performance and lower clogging during spray drying. Finally, physical (scanning electron microscopy-SEM, transmission electron microscopy-TEM, and thermogravimetric analyzer-TGA) and microbiological (mesophiles, yeasts, fungi, and fecal coliforms) characterization is demonstrated. These microparticles are intended to be used in the food industry to produce functional and enriched foods, protecting the bioactive components of interest.

## 1. Introduction

Cellulose has extensive applications in the food industry, being used in baked goods and beverages and as a stabilizing agent, gelling agent, suspending agent, rheological modifier, edible film, colloid laxative, emulsifier, and thickening agent, among others. The use of bacterial nanocellulose (BNC) in food has grown in recent years, but production of bacterial nanocellulose for food applications has been limited, due to the reduction in membrane size caused by processes that use a mixer or a blender. Raw materials used in food have a high standard of quality, and their processing needs to be well controlled from beginning to end [1].

Bacterial nanocellulose has gained interest in the food industry, especially for its purity. Vegetable-derived cellulose (VC) usually exists in the cell wall, forming a complex structure with hemicellulose, lignin, and other substances; therefore, VC requires intensive operations to be processed for use in different foods. Furthermore, both BNC and VC have different chemical and physical features [2,3]. One of the differences lies in the highly ordered structure of these celluloses. With VC, a number of cellulose molecular chains accumulate, forming microfibrils, and subsequently bundles and clusters of highly ordered structures called fibril lamella and fiber cells form. Conversely, the BNC produced by *Komagateibacter medellinensis* is secreted in the form of a ribbon, composed of bundles of microfibrils [4,5]. Such a ribbon is very thin, with a width of only one hundredth that of VC. Ribbon cellulose further grows as a visible reticular structure, forming a regular structure, unlike VC.

BNC has some unique properties when compared with VC, such as high purity, no content of hemicellulose or lignin (present in plant cellulose) [2,6], large water-holding capacity (up to one hundred times its weight) [7], high crystallinity [2,6], excellent biological affinity, a higher Young’s modulus compared with VC (15~30 GPa for BNC-sheets form), and great biodegradability [8]. With the above characteristics, BNC is expected to have applications as a raw material in the food industry. Chemical (using reactions both in aqueous and organic solvents) [9] and mechanical methods (e.g., microfluidization and grinding) [1,10] could be applied to reduce the size of BNC membranes to obtain bacterial nanocellulose (BNC), which is important for producing micro- and nanoparticles.

**Figure 1:**
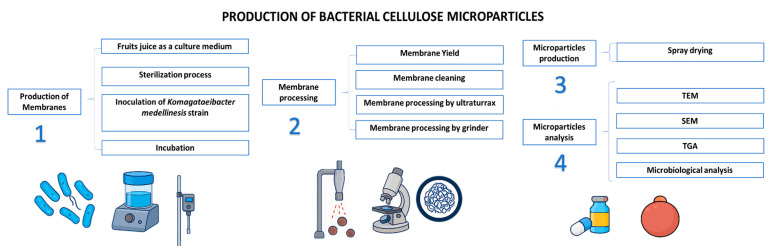
Scheme of production and characterization of microparticles of bacterial nanocellulose.

The main goal of this paper is to propose a whole new process to obtain microparticles of BNC by standardizing the operations from the production of BNC membranes and mechanical processing of BNC membranes to obtain nanocellulose for microparticle synthesis (Figure 1).

Although various studies have explored the production of bacterial nanocellulose (BNC), this research differs by implementing a standardized protocol that integrates low-cost tropical fruit waste as a carbon source, coupled with mechanical treatments optimized for spray drying. Unlike previous reports that often rely on purified sugars or synthetic media [2,11], this approach promotes sustainability and economic feasibility, while preserving the physicochemical integrity of the BNC. Additionally, it is demonstrated how mechanical treatments influence morphological and thermal properties.

## 2. Production of Bacterial Cellulose

Production of bacterial nanocellulose was controlled with the aim to obtain safe membranes for human consumption. The strain used in this study was *Komagataeibacter medellinensis*, and this strain was isolated and purified according to Castro [4]. The following steps describe the correct process to obtain safe bacterial nanocellulose membranes for human consumption.

### 2.1. Obtention and Processing of Culture Medium

In this study, fruits wastes including *Mangifera indica* (mango), *Malus domestica Borkh* (apple), *Ananas sativus* (pineapple), *Carica papaya* (papaya), and *Musa paradisiaca* (banana) and vegetable wastes including *Arracacia xanthorrhiza* (arracacha), *Manihot esculenta* (cassava), and *Zingiber officinale* (ginger) were obtained from the Medellin Archdiocesan Food Bank. These fruits were in the high ripening stage and not suitable for commercial sale. Nonetheless, no rotten fruits were used. These fruits were washed, crushed, squeezed, and separated into juices and residues.

The fruits were blended in a SKYMSEM LAR-04MB (Canelones, Uriguay) for three minutes, two times; after that, the mix was passed through a mesh of different pore diameters to separate solid matter from the liquid. The liquid obtained was standardized to 4 degrees Brix by adding water and to pH 3.5 using acetic acid (optimum pH of *Komagataeibacter medellensis* from Cristina, 2012 [4]). The juice was used as a carbon source (culture medium) for the cultivation of the bacteria, which was previously sterilized at 15 psi for 20 min before inoculation.

The percentage of fruits in the mixture was selected according to the availability of the fruit or vegetable, and the selection of fruit mixtures was initially based on empirical observations of juice yield and contamination risk. The best production of juice was observed in assay 9 (papaya and pineapple), and the lower yield was present in test 2 as is shown in Table 1. It was noted that juices containing banana fruit were becoming dark over time and after the sterilization process even in juices with low percentages of banana.

### 2.2. Sterilization and Disinfection Process

Once the juices were obtained, the bioreactors, working areas, workers, and other elements were either sterilized or disinfected to minimize contamination by foreign microorganisms.

### 2.3. Inoculation

An amount of 1.5 cm^3^ of *Komagataeibacter medellinensis* strain was inoculated onto 8 L of sterilized fruit juice; placed in container of 10 cm height, 20 cm length, and 14 cm width; and incubated at 28 °C for eight weeks until the culture medium was consumed. After the incubation time, the membranes were washed, followed by mechanical processing.

## 3. Bacterial Nanocellulose Membranes

This section describes the transformation process applied to bacterial nanocellulose membranes to reduce their size, enabling them to be passed through a spray dryer to produce microparticles. It also provides information for selecting the optimal fruit mix for producing bacterial nanocellulose membranes and for choosing between two mechanical processing methods (grinding and Ultra-Turrax, Universidad de Antioquia, Medellín, Colombia), based on the fluidity of the bacterial nanocellulose solution for spray drying, with the aim of obtaining microparticles.

### 3.1. Membrane Yield

The membranes’ thickness was around 3 cm and weighed 250–300 gr after two months of incubation. Their moisture content was 98% with 2% dry matter [2] For each test, the yield was calculated by dividing the quantity of membranes obtained by the total juice from each mix. As shown in Table 2, test 9 yielded the best results, followed by test 8, and with test 2 showing the lowest yield. Tests that included vegetables in the mix consistently showed lower yields, whereas fruit-only mixes exhibited high yield. A possible explanation is the type of sugar available in fruits versus vegetables. Bacteria that produce cellulose primary utilize monosaccharides, which are abundant in ripe fruits but are present in lesser amounts in vegetables [12]. Membranes from tests 1, 2, 3, 6, and 7 had a dark color attributed to the browning of banana fruit while tests without banana produced clearer membranes.

### 3.2. BNC Membrane-Washing Process

**Figure 2:**
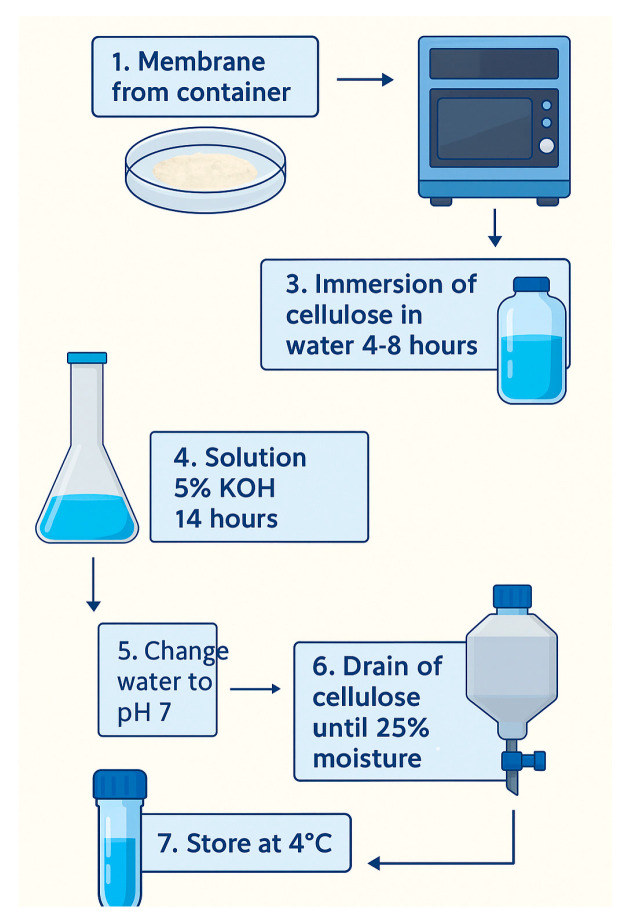
Scheme of the membrane-cleaning process.

The purpose of this step was to remove cellular debris from the culture medium that was trapped in the nanocellulose membranes. At the end of incubation, the nanocellulose membranes were collected and washed with water. The membranes were cut into little pieces manually and then put in a VITA-PREP^®^3 mixer (Olmsted Township, OH, USA) at 6000 rpm followed by immersion in water for 4 to 8 h to eliminate colors and impurities. Then, the samples were washed using 5 wt % KOH aqueous solution, keeping the cellulose immersed for 14 h. Finally, the BNC samples were rinsed with water until they reached pH 7 and filtered by cloth before storage at 4 °C [2]. The membranes from tests 1, 2, 3, 6, and 7 needed to be cleaned up two or more times to remove color; the KOH process including the rinse until pH 7 was repeated as well. The process of washing the membranes is shown in Figure 2.

Test 9, which consisted of a mix of pineapple and papaya, had the best yield (grams of CB membranes/L of juice obtained) with 379.9 g/L; it was also easy to clean up with only one KOH clean-up cycle, reducing cost of production.

### 3.3. Membrane Processing to Reduce Particle Size

Before the spray-drying process, two different methods to reduce the membrane size were used: Ultra-Turrax and grinder. Due to the large size of membranes that were obtained during incubation, it was imperative to process the membranes in a mixer before using an Ultra-Turrax or grinder. The membranes were processed in batches of 200 g of bacterial nanocellulose per liter at 6000 rpm for 5 min, three times in a VITA-PREP^®^3 mixer. During the mechanical processing of bacterial nanocellulose, careful temperature control was crucial to prevent thermal degradation. Both the grinder and Ultra-Turrax systems operated under conditions that kept the suspension below 40 °C throughout the process. This precaution was especially important because nanocellulose is sensitive to elevated temperatures, which can lead to partial hydrolysis or changes in fiber morphology. We continuously monitored the temperature, using intermittent pauses during high-shear mixing to dissipate any accumulated heat. This ensured the structural integrity of the bacterial nanocellulose was preserved before the spray drying and subsequent characterization steps.

#### 3.3.1. Membrane Processing by Ultra-Turrax

**Figure 3:**
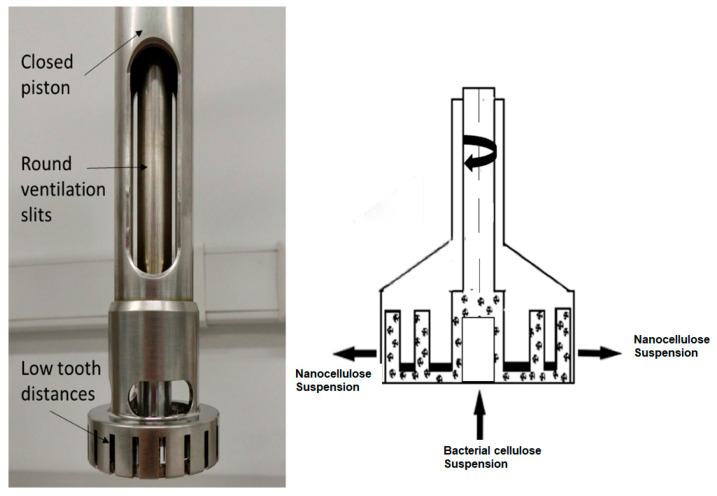
Ultra-Turrax: parts (**left**) and operation (**right**).

Both HSDs (high-speed dispersing machines, Universidad de Antioquia, Medellín, Colombia) and Ultra-Turrax systems are rotor-stator systems like colloid mills (Figure 3). They are composed of coaxial intermeshing rings with radial openings. The fluid enters the center of the system and is accelerated by the rotor. As it passes through the system, the fluid accelerates and decelerates multiple times, which results in high tangential forces. Rotational speeds may be as high as 20,000 rpm.

A solution of 1.5–2.5% wt bacterial nanocellulose was passed through the Ultra-Turrax starting at 3500 rpm and increased to 24,000 rpm. The BNC solution was passed through the Ultra-Turrax three times for no more than five minutes each time to avoid heating the sample. The final solution was used to prepare a second solution of 5% wt bacterial nanocellulose. Upon spray drying the second solution, we observed frequent clogging of the nozzle. This clogging occurred even after the solution had undergone more than three cycles through the Ultra-Turrax, suggesting that further size reduction beyond three cycles was not attainable.

#### 3.3.2. Membrane Processing by Grinder

**Figure 4:**
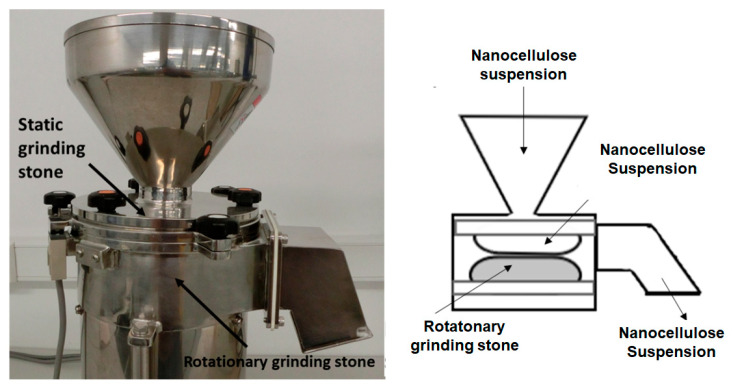
Grinder: parts (**left**) and operation (**right**).

Nowadays, grinding is one of the most common methods, as it is not hindered by clogging issues and it can improve the efficiency of the fibrillation process. Its operation is based on the shear stress imposed by two non-porous ceramic discs (Figure 4), one rotating at speeds between 750 and 3000 rpm, while the other is kept stationary [13,14].

A reduction in size of the nanocellulose membrane was made using a Masuko Sangyo MKCA6-2 grinder (Kawaguchi-city, Japan) with a MK GC 6–120 SD disc. Five different sizes in the gap between the grinder discs were used to homogenize the nanocellulose, passing it five times per each gap between the discs. The cellulosic material at a concentration of 2% wt was passed 25 times through the grinder equipment. The gaps between discs were 1, 0.5, 0.0, −0.5, and −1.

The bacterial nanocellulose processed by the grinder had a creamy consistency and was used to prepare a second solution at 5% (*v*/*w*) bacterial nanocellulose. When this second solution was spray dried, a fluid movement of the solution through the nozzle was observed, with no clogging.

### 3.4. SEM Analysis of Bacterial Nanocellulose Obtained by Ultra-Turrax and Grinder

**Figure 5:**
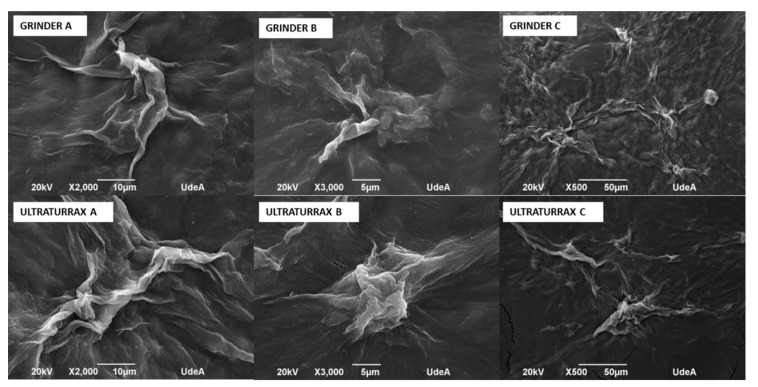
Micrographies of bacterial nanocellulose treated by grinder and Ultra-Turrax.

Five solutions with different concentrations of bacterial nanocellulose were prepared: 5, 2.5, 1.25, 0.625, and 0.312% *w*/*v* for both types of mechanical processing: Ultra-Turrax and grinding. A drop of each solution was deposited onto carbon tape and dried at room temperature. Morphological features of bacterial nanocellulose from Ultra-Turrax and grinding after a pass by spray drying were determined by scanning electron microscopy (SEM) (Hefei, Anhui, China) using a JEOL JSM 6490 LV at high vacuum operated at an acceleration voltage of 20 kV. The samples were covered with sputtered gold before observation. The size distribution of fibers of bacterial nanocellulose processed by both the grinder and Ultra-Turrax was determined using the software ImageJ images and is shown in Figure 5.

It is possible to observe the agglomerates of bacterial nanocellulose. More and bigger agglomerations were observed in Ultra-Turrax images than those of the grinder at the same magnifications.

**Figure 6:**
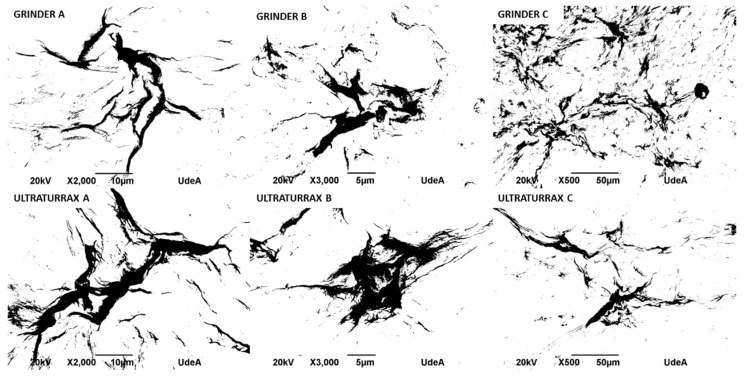
Micrographs of bacterial nanocellulose, treated by grinding and Ultra-Turrax, processed by ImageJ.

The image analysis presented in Table 3 was performed using ImageJ software version 1.53, following the procedures recommended by Hartig [15]. Each micrograph (Figure 6) was first converted to 8-bit grayscale and then processed by applying a thresholding technique to isolate regions corresponding to bacterial nanocellulose agglomerates. The threshold was adjusted to distinguish darker areas (indicative of cellulose clusters) from the background, allowing for consistent segmentation across images. Following thresholding, the “Analyze Particles” function in ImageJ was used to extract key morphological parameters. The total area (µm^2^) represents the absolute area occupied by the agglomerates, while the %area denotes the proportion of the image occupied by these agglomerates. The perimeter (µm) parameter was used to quantify the total boundary length of each detected agglomerate, serving as an indicator of shape complexity and spatial extent. The integrated density (AU), defined as the product of the area and the mean gray value of each agglomerate [11], was calculated to provide a composite measure of both size and intensity of the cellulose clusters. This parameter helps us infer the concentration and compactness of the bacterial nanocellulose structures. As shown in Table 3, the Ultra Turrax-treated samples generally exhibited higher total area, perimeter, and integrated density values compared to the grinder-treated samples for samples A and B, suggesting a more extensive agglomeration behavior under Ultra-Turrax conditions. Conversely, sample C displayed greater agglomeration in the grinder condition, though Ultra-Turrax led to larger but less-dispersed clusters. These findings highlight the influence of mechanical treatment on the dispersion and structural organization of bacterial nanocellulose.

The grinding process produced BNC solutions that were less agglomerated, which allowed better fluidity through the spray equipment, and a solution prepared with bacterial nanocellulose that did not clog in the pipe. So, the grinder was selected to process bacterial nanocellulose and produce microparticles of bacterial nanocellulose by the spray-drying method.

## 4. Microparticles by Spray Drying

### 4.1. Production

Before the spray-drying process, the concentration of bacterial nanocellulose in suspension was verified by gravimetric analysis. A known volume of the nanocellulose suspension was dried at 105 °C until constant weight, and the remaining dry mass was used to calculate the solid content, expressed as weight/volume percentage (*w*/*v*%). This procedure ensured reproducibility in the microencapsulation process by standardizing the amount of nanocellulose present in each batch. Maintaining consistent nanocellulose concentration was essential to produce microparticles with comparable morphology, as later evaluated through TEM (Transmission Electron Microscopy) and SEM (Scanning Electron Microscopy) imaging, and to assess the impact of concentration on encapsulation efficiency and thermal behavior observed in TGA analysis (Thermogravimetric Analyzer).

Microparticles of bacterial nanocellulose were produced by using a Buchi B-290 mini spray dryer with a standard 0.5 mm nozzle under the follow conditions (selected considering preliminary studies): dispersion (5 mL/min (25%)) and air flow rates (35 m^3^/h (90%)), air pressure (6.0 bar), and inlet and outlet temperature (200 °C and 100 °C) with a standard 0.5 mm nozzle. A solution of 0.5% of bacterial nanocellulose was prepared under constant stirring at 1000 rpm for 20 min using a Young Ji HMZ 20DN stirrer (Hana Instruments); the stirring was maintained while the solution was passed through the spray dryer. When the liquid was fed into the nozzle with a peristaltic pump, atomization occurred due to the force of the compressed air, disrupting the liquid into small droplets. The droplets, together with hot air, were blown into a chamber where the solvent in the droplets was evaporated and discharged through an exhaust tube. The resulting dry powder was collected in a receiving flask, with a particle recovery yield of approximately 60% ± 3%, calculated based on the initial solid content in the feed solution.

### 4.2. Analysis of Microparticles

Characterization of these microparticles was performed by TEM, SEM, and TGA, and microbiological analysis was carried out. TEM and SEM were used to morphologically analyze each. TGA was used to determine if the maximum degradation temperature of bacterial nanocellulose changes between its membrane and microcapsule forms. Finally, microbiological analysis was conducted to define the long-term stability of the bacterial nanocellulose powder. These analyses are detailed below.

#### 4.2.1. TEM (Transmission Electron Microscopy)

TEM was used to observe bacterial nanocellulose suspensions and spray-dried microparticles, following the procedure outlined by Castro [4]. After a brief sonication to achieve a good dispersion, drops of diluted bacterial nanocellulose suspensions were deposited onto glow-discharged, carbon-coated TEM grids and negatively stained with 2% wt uranyl acetate. Samples were observed using a Philips CM200 (Leuven, Belgium) microscope operating at 200 kV. The images were recorded with a TVIPS TemCam F216 camera (Leuven, Belgium).

**Figure 7:**
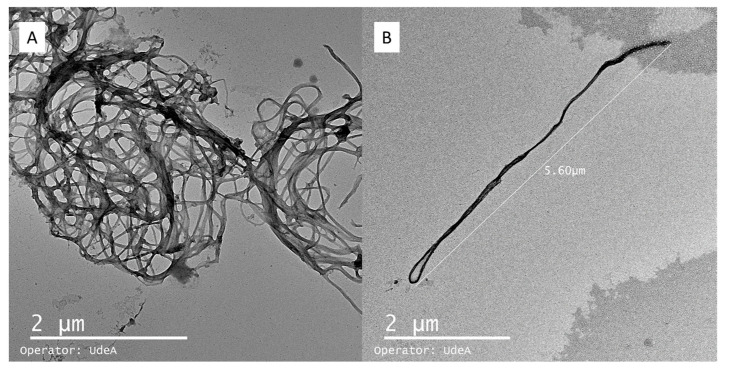
Microparticles of bacterial nanocellulose observed by TEM (Transmission Electron Microscopy). Bacterial nanocellulose agglomerations (**A**); isolated fiber of bacterial nanocellulose (**B**).

TEM analysis is shown in Figure 7. The micrograph in Figure 7A reveals a fibrillar network structure composed of bacterial nanocellulose nanoribbons. These nanoribbons exhibit a typical width ranging from 30 to 50 nm and appear entangled, forming a porous mesh. In Figure 7B, an individual nanoribbon is clearly distinguished, with an estimated length of approximately 7–8 µm, which aligns with previous reports for bacterial nanocellulose fibers in aqueous dispersion. Measurements were conducted on at least 50 nanoribbons across multiple fields of view to ensure representative analysis. The average fiber diameter was 42 ± 6 nm, indicating a relatively homogeneous nanoscale morphology.

Notably, the observed nanostructures are not compact or solid nanoparticles; instead, they consist of flexible, high-aspect-ratio fibrils. This morphology suggests that the material is more suitable as a structural matrix or encapsulating scaffold rather than as discrete spherical particles. The extended and interconnected network observed confirms the potential of bacterial nanocellulose for forming microcapsules via entanglement rather than classical nanoparticle aggregation.

#### 4.2.2. SEM (Scanning Electron Microscopy)

**Figure 8:**
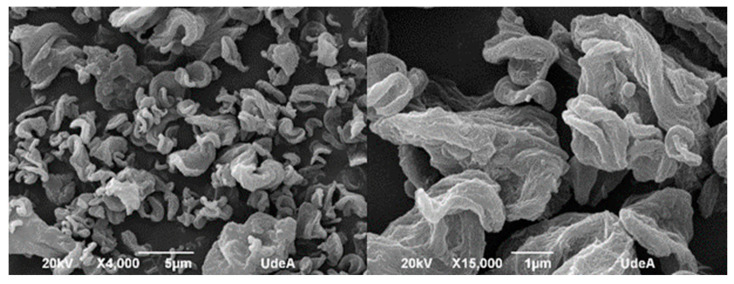
Micrographies of bacterial nanocellulose microparticles (Scanning Electron Microscopy).

Morphological features of bacterial nanocellulose microparticles were determined by scanning electron microscopy (SEM) by using a JEOL JSM 6490 LV at high vacuum operated at an acceleration voltage of 20 kV. A powder sample was deposited onto carbon tape and coated with sputtered gold before observation. SEM analysis of the microparticles is shown in Figure 8. The size distribution of bacterial nanocellulose microparticles was determined using ImageJ software, based on measurements of 100 particles randomly selected from high-resolution SEM images. The particle diameters ranged from a minimum of 2.5 µm to a maximum of 7.26 µm, with a mean particle size of 4.76 ± 1.12 µm. The most frequent particle size fell within the 4–5 µm range, as observed in the distribution histogram.

At 15,000× magnification, the surface of the microparticles appeared rough and irregular, which can be attributed to the interweaving of nanoribbons forming a three-dimensional network. This morphology supports the idea that during the spray-drying process, the bacterial nanocellulose fibers tend to aggregate into microscale spheres, driven by surface tension and drying kinetics. Consequently, most microparticles exhibit a quasi-spherical geometry, although many show irregularities and surface porosity.

Show in SEM analysis, the rough and porous surface of microparticles has potential advantages in encapsulation efficiency and controlled-release properties [10,13]. The tendency of BNC to form quasi-spherical agglomerates supports its use in flowable powders, which are confirmed by clog-free spray drying. Despite this tendency toward spherical formation, the overall shape of the spray-dried particles is amorphous, indicating incomplete or uneven consolidation of the nanoribbons. This morphological behavior suggests that bacterial nanocellulose has potential as a structural encapsulating agent, particularly for systems requiring porosity or controlled-release characteristics. The structural roughness may also favor compound retention within the web-like architecture of the microparticles.

#### 4.2.3. TGA (Thermogravimetric Analysis)

Thermogravimetric analysis was carried out using 10 mg of dried filtrate cake obtained from the vitamin/BNC mixture, previously filtered and dried. Analyses were performed on a Mettler Toledo TGA/SDTA 851e (Greifensee, Switzerland) instrument under a nitrogen atmosphere (40 mL·min^−1^), with a heating rate of 10 °C·min^−1^ from 30 °C to 800 °C.

**Figure 9:**
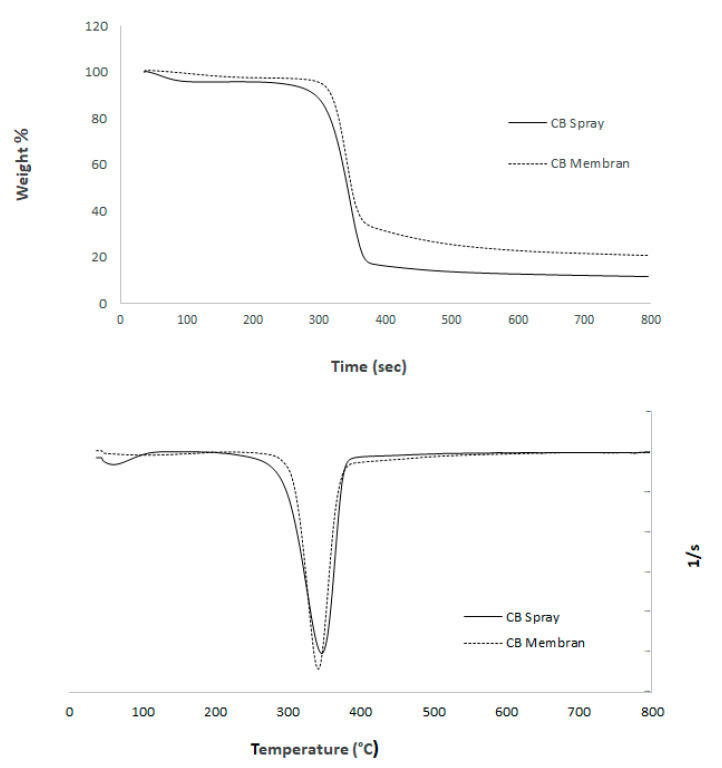
Thermogram of bacterial nanocellulose membranes and microparticles obtained by spray drying: DTG curve (**bottom**); TGA curve (**top**).

As shown in Figure 9, the thermograms of both spray-dried microparticles and membrane-formed bacterial nanocellulose exhibited similar thermal stability profiles. The maximum degradation temperature (T_max) for the BNC membranes was observed at 342 °C, while that of the BNC microparticles was slightly higher, at 345 °C. The onset of thermal degradation occurred at approximately 270 °C for both samples, and total mass loss by 800 °C was 89.6% for membranes and 90.1% for microparticles, indicating a comparable organic content and thermal behavior.

The minimal difference in T_max (<1%) suggests that the spray-drying process did not alter the crystallinity or thermal resistance of the bacterial nanocellulose. This observation is consistent with the fact that both processing methods preserved the molecular integrity of the nanofibers. Additionally, the DTG curves (Figure 9, left) show a single prominent degradation peak for both samples, centered around 340–345 °C, further confirming the structural stability of the material regardless of drying technique. The DTG analysis revealed a single, well-defined degradation peak for both bacterial nanocellulose (BNC) membranes and spray-dried microparticles, centered around 345 °C, which corresponds to the main pyrolytic decomposition of cellulose. This peak is associated with the breakdown of glycosidic linkages and the depolymerization of cellulose chains, a process typically observed in high-purity nanocellulose [10]. The absence of multiple degradation peaks or shoulder regions suggests that the samples contain negligible amounts of hemicellulose, lignin, or residual biomass contaminants, confirming the effectiveness of the purification and mechanical processing steps [4,11]. Notably, no significant shift was observed between the degradation temperatures of membranes (342 °C) and microparticles (345 °C), indicating that spray drying did not alter the crystalline domains or disrupt the thermal stability of the nanofibers. This thermal behavior implies that inter- and intramolecular hydrogen bonding in the cellulose structure was preserved, which is critical for applications requiring structural integrity under processing or storage conditions. Furthermore, the sharpness of the DTG peak reflects a narrow degradation range, consistent with a homogeneous material phase. These findings support the suitability of BNC microparticles for thermally stable delivery systems in food and pharmaceutical applications.

Compared to conventional encapsulating agents such as maltodextrin and alginate, the bacterial nanocellulose (BNC) microparticles produced in this study offer distinct structural and functional advantages. Traditional carriers are often selected for their solubility and ease of processing; however, they typically exhibit limited mechanical strength, lower thermal stability, and reduced barrier properties. In contrast, BNC microparticles demonstrated high thermal resistance (up to 345 °C), robust structural integrity, and a fibrous network morphology that provides a dense, entangled matrix suitable for controlled-release applications. Moreover, BNC’s high crystallinity and nanofibrillar architecture contribute to excellent mechanical properties and moisture-barrier capacity, which are critical for protecting sensitive bioactive compounds [16,17]. These features suggest that BNC is not only a viable but also a potentially superior alternative to conventional encapsulants in applications demanding structural stability and long-term preservation.

#### 4.2.4. Shelf Life of Bacterial Nanocellulose Microparticles

Moisture, Aw, and microbiological analyses were conducted on bacterial nanocellulose powder over 90 days to determine the stability of the microparticles. Principles and guidelines for the establishment and application of microbiological criteria related to foods were made according to codex alimentarius CAC/GL-21, 1997 [18] and ISO 7218 [19]. The sampling was performed using the general guidelines of sampling according with CAC/GL 50-2004 [20] international parameters. Aerobic colony count (ACC) was considered satisfactory with <104 colony-forming unit (cfu)/g. The limit of mesophiles in powder is 106 (U.F.C/g); for yeast and molds the limit is set in <200 (U.F.C/g) and fecal coliforms <1. Dry matter and moisture content were analyzed using a Shimadzu MOC 63u moisture balance, and water activity (Aw) was measured with a Rotronic Hygropalm HP23-AW-A meter.

Before submitting the samples to analysis, the samples were stored under extreme conditions set at 40 °C and 75% RH at four different times: 0, 30, 60, and 90 days (Table 4). Microbiological results showed no growth of fungi, fecal coliforms, and total coliforms. These results were expected due to the low Aw of 0.9 that does not allow for the growth of these microorganisms. On the other hand, regarding the presence of mesophiles and yeast, microbiological analyses were conducted on bacterial nanocellulose powder over a period of 90 days to determine the stability of the microparticles, which was expected due to the fact that the incubation temperature was appropriate to the growth of these microorganisms. There was a rise in the counts of mesophiles, yeast, moisture, and Aw over the 90 days. Nonetheless, mesophiles remained under the previously mentioned allowed limits according to the international regulation for powders.

The final microparticles obtained from bacterial nanocellulose (BNC) exhibited promising physicochemical and structural properties that support their potential use as encapsulating agents. The particles presented a mean diameter of 4.76 ± 1.12 µm, a rough but cohesive surface morphology, and thermal stability up to 345 °C, all of which indicate good resistance to processing conditions. In addition, low moisture content (<10%) and stable water activity (Aw < 0.65) suggest microbiological stability and shelf-life potential comparable to industrial standards. The microparticles also maintained the nanofibrillar structure of BNC, allowing for porosity and potential controlled-release applications.

## 5. Conclusions

This study successfully established a standardized and sustainable method for producing bacterial nanocellulose (BNC) microparticles using fruit-based substrates and mechanical treatments, followed by spray drying. Among the mechanical methods tested, the grinder treatment demonstrated superior performance by generating a more uniform dispersion of nanofibers, which reduced clogging during spray drying and produced particles with consistent morphology and size distribution. SEM and TEM analyses revealed that the resulting microparticles maintained a porous, fibrous structure with an average particle size of 4.76 ± 1.12 µm, favoring potential applications in encapsulation systems.

Thermogravimetric analysis showed that both BNC membranes and microparticles retained high thermal stability, with maximum degradation temperatures of 342 °C and 345 °C, respectively. This indicates that the spray-drying process did not alter the structural integrity or crystallinity of the nanofibers. Moreover, the low moisture content and stable water activity (Aw < 0.65) ensured microbiological safety over a 90-day storage period, confirming the shelf-life potential of the powdered product. Compared to conventional encapsulating agents, BNC offers advantages in mechanical strength, barrier properties, and thermal resistance, positioning it as a promising biomaterial for controlled-release and protective delivery systems in food and pharmaceutical applications. Future work should focus on encapsulation efficiency and release kinetics of active compounds to validate the microparticles in real-use conditions.

From a regulatory perspective, bacterial nanocellulose has shown compatibility with several international frameworks. In food applications, BNC may be considered under the GRAS criteria regulated by the U.S. Food and Drug Administration [21], while the European Food Safety Authority (EFSA) outlines specific guidance on nanomaterials in food [22]. For biomedical uses, the material aligns with ISO 10993 [23] for biocompatibility, and its morphological and thermal properties support its potential for encapsulation and controlled-release systems, as discussed in ICH Q8–Q10 guidelines [24]. Integrating these normative considerations strengthens the scientific basis of the study and highlights the suitability of BNC microparticles for industrial implementation in safe and standardized conditions.

While a direct quantitative comparison with commercially available microencapsulation systems (e.g., maltodextrin-, alginate-, or gelatin-based carriers) was not conducted in this study, the thermal and morphological characteristics of the BNC microparticles are within the range reported for these traditional matrices. Moreover, BNC offers added advantages such as biodegradability, non-toxicity, and mechanical strength, which are highly desirable in food and pharmaceutical applications [16,25]. Future studies should include comparative performance tests, including encapsulation efficiency, release kinetics, and in vitro digestion models, to more accurately benchmark the BNC microparticles against existing commercial products.

## Figures and Tables

**Table 1:** Mixes of fruits used as culture medium of *Komagataeibacter medellinensis*.

Assay	Mix of Fruits/Vegetables	% of Fruits in the Mix	% of Juice (L Juice/kg Fruits)
Assay 1	Pineapple, mango, tangerine, papaya, granadilla passion fruit, banana	20, 15, 15, 15,15, 15	82
Assay 2	Arracacha, cassava, ginger, banana, mango	10, 10, 10, 35, 35	52.5
Assay 3	Tree tomato, mango, banana	40, 30, 30	75
Assay 4	Mango, papaya, apple	40, 40, 20	100
Assay 5	Pineapple, mango	50, 50	128
Assay 6	Mango, banana	50, 50	130
Assay 7	Papaya, banana	50, 50	130
Assay 8	Mango, papaya, pineapple	30, 30, 40	200
Assay 9	Papaya, pineapple	50, 50	211

Number of replicates for each experimental stage (n = 5).

**Table 2:** Membrane yield from bacterial nanocellulose.

Assay	Juice (L)	Membrane Weight (kg)	Yield (g/L)
1	22	4531	206.0
2	50	6988	139.8
3	30	9957	331.9
4	41	9861	240.5
5	52	16,943	325.8
6	52	17,487	336.3
7	50	16,734	334.7
8	55	20,198	367.2
9	53	20,136	379.9

Number of replicates for each experimental stage (n = 5).

**Table 3:** Data of bacterial nanocellulose treated by grinder and Ultra-Turrax obtained by ImagJ program.

Image	Total Area (µm^2^)	%Area	Perimeter (µm)	Integrated Density (AU)
Grinder A	1.591	8.355	0.091	0.289
Ultra-Turrax A	2.819	14.802	0.127	0.612
Grinder B	1.662	8.728	0.093	0.336
Ultra-Turrax B	2.540	13.334	0.124	0.778
Grinder C	3.085	16.196	0.069	0.143
Ultra-Turrax C	1.175	6.167	0.077	0.180

**Table 4:** Parameters used to determine the shelf life of bacterial nanocellulose powder.

Time (Days)	Mesophiles (U.F.C/g)	Yeasts (U.F.C/g)	Fungi	Fecal Coliforms	Total Coliforms	Moisture (%)	Aw
0	193	5	0	0	0	9	0.458
30	293	100	0	0	0	9.34	0.54
60	301	100	0	0	0	9.74	0.626
90	249	168	0	0	0	15.24	0.622

## Data Availability

The original contributions presented in this study are included in the article. Further inquiries can be directed to the corresponding author.
